# Earlier smoking after waking and the risk of asthma: a cross-sectional study using NHANES data

**DOI:** 10.1186/s12890-018-0672-y

**Published:** 2018-06-18

**Authors:** Arielle S. Selya, Sunita Thapa, Gaurav Mehta

**Affiliations:** 1 Master of Public Health Program, Department of Population Health, University of North Dakota, 1301 North Columbia Rd. Stop 9037, Grand Forks, ND 58202 USA; 2 Department of Public Policy, Vanderbilt University School of Medicine, 2525 West End Ave, Suite 1200, Nashville, TN 37203 USA

**Keywords:** Asthma, Cigarettes, Nicotine dependence, Smoking, Time to first cigarette

## Abstract

**Background:**

Recent research shows that nicotine dependence conveys additional health risks above and beyond smoking *behavior*. The current study examines whether smoking within 5 min of waking, an indicator of nicotine dependence, is independently associated with asthma outcomes.

**Methods:**

Data were drawn from five pooled cross-sectional waves (2005–14) of NHANES, and the final sample consisted of *N* = 4081 current adult smokers. Weighted logistic regressions were run examining the relationship between smoking within 5 min of waking and outcomes of lifetime asthma, past-year asthma, and having had an asthma attack in the past year. Control variables included demographics, smoking behavior, family history of asthma, depression, obesity, and secondhand smoking exposure.

**Results:**

After adjusting for smoking behavior, smoking within 5 min was associated with an approximately 50% increase in the odds of lifetime asthma (*OR* = 1.46, *p* = .008) and past-year asthma (*OR* = 1.47, *p* = .024), respectively. After additionally adjusting for demographics and other asthma risk factors, smoking within 5 min of waking was associated with a four-fold increase in the odds of lifetime asthma (*OR* = 4.05, *p* = .015).

**Conclusions:**

Smoking within 5 min of waking, an indicator of nicotine dependence, is associated with a significantly increased risk of lifetime asthma in smokers. These findings could be utilized in refining risk assessment of asthma among smokers.

**Electronic supplementary material:**

The online version of this article (10.1186/s12890-018-0672-y) contains supplementary material, which is available to authorized users.

## Background

Asthma is a highly disabling but treatable disease, which consists of chronic airway inflammation and episodes of acute exacerbations in response to certain stimuli. Several risk factors for asthma have been identified, and a notable avoidable risk factor is cigarette smoking [1]. However, most existing research does not distinguish between nicotine dependence (ND), a psychological construct capturing the addiction to nicotine, and smoking *behavior*. Traditionally, smoking behavior and ND were largely considered synonymous; however, recent research has increasingly highlighted the importance of distinguishing between them. ND is a psychological construct capturing addiction to smoking, and this is to some extent separable from smoking behavior itself. For example, adolescent smokers often develop ND at low, even non-daily levels of smoking [2–4], and this predicts future smoking behavior even after accounting for prior smoking behavior [3, 5, 6], while other demographic groups show very low ND despite heavy smoking behavior [2]. Moreover, ND poses a higher risk than does smoking behavior alone for several other smoking-related health outcomes, including lung cancer [7, 8], larynx cancer [9], head and neck cancer [10], and chronic obstructive pulmonary disease (COPD) [11, 12]. A limited number of studies reported that asthma significantly increases the risk of both smoking behavior and ND [13, 14]; however, the association between asthma and ND was not assessed *independently* of smoking behavior. Thus, very little is known about whether higher ND is associated with increased risk of asthma, over and above smoking behavior.

The current study tests the novel hypothesis that smoking within 5 min of waking, a version of time to first cigarette (TTFC) which is a strong and reliable indicator of ND [15, 16], is associated with a greater risk of asthma even after accounting for lifetime smoking history. Data are pooled across five consecutive, cross-sectional waves (2005–06, 2007–08, 2009–10, 2011–12, and 2013–14) of the National Health and Nutrition Examination Survey (NHANES) survey, and include current adult smokers. Though causality cannot be established here, data were restricted to those who initiated smoking prior to developing asthma, in order to ensure a temporal relationship that is at least *consistent with* smoking as a causal factor in the development or exacerbation of asthma. The relationships between smoking within 5 min after waking and binary outcomes of 1) lifetime asthma, 2) past-year asthma, and 3) having had an asthma attack in the past year were examined using weighted logistic regressions. Control variables included smoking behavior (past-month cigarettes per day (CPD) and years of smoking duration), demographic characteristics (age, race/ethnicity, and gender), and other risk factors for asthma (obesity, family history of asthma, depression, and secondhand smoke exposure).

## Methods

### Study sample

This study utilized data from five cross-sectional waves (2005–06, 2007–08, 2009–10, 2011–12, and 2013–14) of NHANES, a publicly available, nationally-representative survey conducted by the Centers for Disease Control. NHANES is a nationally representative sample of the non-institutionalized civilian U.S. population that is conducted on an ongoing, semiannual basis since 1999. NHANES consists of an extensive questionnaire on medical conditions, health-related behaviors and risk factors, and a subcomponent involving physical examination and laboratory tests. Since complete smoking behavior and ND information was available only from current smokers 20 years or older, participants younger than 20 or who did not report being a current smoker were excluded from all analyses. Additionally, among participants who reported having had asthma at some point in their lifetime, those who developed asthma prior to initiating smoking were also excluded. The final analytic sample includes *N* = 4081 current adult smokers.

### Measures

Asthma outcomes were examined with three binary variables: self-reported lifetime asthma (“Has a doctor or other health professional ever told you that you have asthma?”); and among those who said yes, self-reported past-year asthma (“Do you still have asthma?”), and having reported at least one asthma attack in the past year (“During the past 12 months, have you had an episode of asthma or an asthma attack?”). Additionally, self-reported age of asthma diagnosis (“How old were you when first told you had asthma?”) was used to determine exclusion from the final study sample: participants were excluded if age of asthma diagnosis was younger than the self-reported age of first regular smoking.

Smoking within 5 min after waking was derived from self-reported time to first cigarette (TTFC), perhaps the best single-item measure of ND [15, 16], which was asked using the question “How soon after waking up do you smoke?” In order to ensure sufficient group sizes in the current sample, the four original response categories were dichotomized into ≤5 (high dependence) vs. > 5 min (low to moderate dependence), consistent with other recent research [17].

Control variables included current smoking behavior (self-reported cigarettes per day (CPD) in the past 30 days) and lifetime smoking behavior (years of smoking duration, calculated by subtracting self-reported age of first regular smoking from age at questionnaire), in accordance with recent recommendations for controlling for smoking behavior [18] .

Additionally, demographic characteristics included age, sex, and race/ethnicity. Race/ethnicity was self-reported as “non-Hispanic white,” “non-Hispanic black,” “Mexican-American,” “other Hispanic,” and “other;” for the current analyses, “Mexican-American” and “other Hispanic” were combined into a single category, and participants reporting “other” were excluded from analyses due to small sample size (*N* = 220).

Other risk factors for asthma were used as potential confounding variables, and included self-reported family history of asthma, depression (derived from 10 items based on the DSM-5 criteria), obesity (measured in the physical examination component of NHANES), and secondhand smoke exposure (SHS; dichotomized into any vs. no self-reported exposure to cigarette smoke at home and in the workplace).

### Statistical analysis

Weighted logistic regression was used in accordance with NHANES Analytic Guidelines [19] using R statistical software and its “survey” package to examine outcomes of lifetime asthma, past-year asthma, and having had an asthma attack in the past year. Weighted regressions take into account NHANES’ complex sampling design to produce results that are representative of the larger U.S. noninstitutionalized civilian population, rather than the particular NHANES sample. The outcomes were each examined as a function of smoking within 5 min after waking. For each outcome, three models of varying complexity were run: 1) an unadjusted model; 2) a model adjusted for smoking behavior (past-month CPD and years of smoking duration); and 3) a model additionally adjusted for other risk factors for asthma (family history of asthma, depression, obesity), demographic characteristics (age, race/ethnicity, and sex), and interactions of TTFC with a) secondhand smoke exposure [20, 21] and b) sex [22]. Missing data on variables were handled by listwise deletion.

## Results

Table 1 shows descriptive statistics separately for those who smoke within 5 min of waking (*N* = 1290) and those who smoke after 5 min of waking (*N* = 2791). Those who reported smoking within 5 min of waking were approximately 1.5 times as likely to report having asthma at some time in their lives, 1.5 times as likely to report still having asthma in the past year, and 1.7 times as likely to report having had an asthma attack in the past year. Additionally, those who smoked within 5 min after waking reported heavier past-month smoking, and a longer lifetime smoking duration.

**Table 1:** Descriptive statistics of sample

Measure	Smoking within 5 min of waking (*N* = 1290)	Smoking after 5 min of waking (*N* = 2791)	*p*
Lifetime asthma	**150, 11.6%**	**210, 7.5%**	**<.001**
Past-year asthma	**115, 9.0%**	**153, 5.5%**	**<.001**
Past-year asthma attack	**60, 4.8%**	**82, 3.0%**	**.006**
Cigarettes per day	**20.0, 1.0–25.0**	**10, 6.0–20.0**	**<.001**
Smoking duration	**29.5, 18.0–40.0**	**28, 15.0–40.0**	**.001**
Age	46.0, 35.0–56.0	46.0, 33.0–59.0	.969
Sex			.753
Female	561, 43.5%	1230, 44.1%	
Male	729, 56.5%	1561, 55.9%	
Race			**<.001**
White	**812, 63.0%**	**1541, 55.2%**	
Black	**352, 27.3%**	**698, 25.1%**	
Hispanic	**126, 9.8%**	**552, 19.8%**	
Depression	**261, 23.5%**	**388, 16.0%**	**<.001**
Obesity	371, 30.4%	817, 30.8%	.843
Family history of asthma	**324, 25.8%**	**575, 21.1%**	**.001**
Secondhand smoking	**895, 92.8%**	**1460, 75.9%**	**<.001**

Categorical variables are presented as *N*, valid percentage, and continuous variables are presented as median, interquartile range. *p*-values are based on chi-square tests for categorical variables, and Wilcoxon signed-rank tests for continuous variables. Bold: *p* < .05

Weighted regression analyses of varying complexity are presented in Table 2. At the unadjusted level, smoking within 5 min of waking was associated with higher odds of lifetime and past-year asthma, as well as of a past-year asthma attack. When adjusting for current and lifetime smoking behavior, smoking within 5 min of waking was associated with an approximately 47% increase in the odds of lifetime asthma, and a 46% increase in the odds of past-years asthma. Finally, when also adjusting for demographic characteristics and other risk factors for asthma, smoking within 5 min of waking remained independently associated with lifetime asthma, such that those who smoke within 5 min of waking have approximately four times the odds of reporting lifetime asthma. Secondhand smoke exposure significantly moderated this relationship, such that SHS exposure slightly weakened the effect of smoking within 5 min after waking. Finally, there was a trend between smoking within 5 min of waking with both past-year asthma and having had an asthma attack in the past year. Unweighted regressions are presented in Additional file 1: Table S1.

**Table 2:** Weighted regression results of smoking within 5 min of waking on asthma outcomes

Model	Covariate	Outcome		
		Lifetime Asthma	Past-Year Asthma	Past-Year Asthma Attack
		OR (95% CI) *p-* value	OR (95% CI) *p-*value	OR (95% CI) *p* value
Unadjusted	Smoking within 5 min. (vs. > 5 min.)	**1.71 (1.26–2.31)** ***p*** **= .001**	**1.72 (1.22–2.43)** ***p*** **= .003**	**1.66 (1.04–2.67)** ***p*** **= .038**
Adjusted for smoking behavior^a^	Smoking within 5 min. (vs. > 5 min.)	**1.47 (1.11–1.93)** ***p*** **= .008**	**1.46 (1.06–2.01)** ***p*** **= .024**	1.31 (0.82–2.09) *p* = .268
Adjusted for smoking^a^ and other covariates^b^	Smoking within 5 min. (vs. > 5 min.)	**4.05 (1.35–12.14)** ***p*** **= .015**	3.95 (0.93–16.84) *p* = .069	5.45 (0.85–34.88) *p* = .079
	Smoking within 5 min x SHS	**0.23 (0.08–0.71)** ***p*** **= .014**	**0.20 (0.04–0.90)** ***p*** **= .041**	0.18 (0.02–1.31) *p* = .096
	Smoking within 5 min x sex	**–**	**3.21 (1.56–6.64)** ***p*** **= .003**	–

Results are presented as odds ratio (95% confidence interval), *p*-value. Boldface indicates statistical significance (*p* < .05)

^a^ Smoking covariates: cigarettes per day and years of smoking duration

^b^ Other covariates: depression, obesity, family history of asthma, secondhand smoke exposure (SHS), age, sex, race/ethnicity, and interactions of smoking within 5 min with secondhand smoke exposure and sex. Interaction terms are only included in the model if *p* < .10

Significant interactions were also found between smoking within 5 min of waking and secondhand smoke exposure in the fully-adjusted model for both lifetime and past-year asthma (Table 2). This indicates significant moderation, such that the odds of asthma for those with both risk factors (smoking within 5 min of waking and being exposed to second hand smoke) are lower than would be expected based on their respective independent OR’s. Similarly, an additional interaction between smoking within 5 min of waking and sex was found only for outcomes of past-year asthma, such that being male and smoking within 5 min of waking results in a *higher* risk of past-year asthma than would be expected by the respective independent OR’s of these variables.

## Discussion

This study presents the novel finding that smoking sooner after waking, a reliable indicator for ND, is independently associated with a heightened risk of lifetime and recent asthma outcomes among a nationally representative sample of current adult smokers, over and above current and lifetime smoking behavior. Notably, the association with lifetime asthma remained significant after also adjusting for demographic characteristics and other risk factors for asthma, such that those who smoke within 5 min of waking are at an approximately four-fold risk of reporting asthma at some point in their lives.

The relationships between smoking within 5 min of waking with outcomes of past-year asthma and past-year asthma attacks were not significant after adjusting for current and lifetime smoking behavior, demographics, and other risk factors for asthma. Considering that active smoking has been shown to aggravate inflammation and hypersensitivity of the airways [23], we had expected to find that smoking within 5 min of waking to be associated with past-year asthma outcomes as well. However, the relationship did show a trend (*p* < .10), which suggests that this negative finding may reflect low statistical power due to a relatively small analytic sample given the complexity of the fully-adjusted model. This possibility is bolstered by the fact that smoking within 5 min was associated with both past-year asthma and past-year asthma attacks at the bivariate level, and with past-year asthma after adjusting only for current and lifetime smoking behavior. Future studies using larger sample sizes and/or prospective data are needed to more rigorously evaluate whether indicators of ND are associated with concurrent asthma outcomes and the exacerbation of asthma symptoms.

It is noteworthy that the strength of the relationship between smoking within 5 min and asthma was stronger *after* adjusting for covariates, relative to the unadjusted model and the model adjusted only for smoking behavior. Including the interaction terms of smoking within 5 min with secondhand smoke exposure and with sex were critical in explaining this increase; excluding these significant interactions resulted in substantially lower and even nonsignificant OR’s (data not shown). The significance of these interaction terms indicate important moderation of the relationship between time to first cigarette in the morning and asthma. In the case of sex, the current findings align with sex difference in the relationship between ND and asthma [22]; though differences in the statistical models prevent a direct comparison with the current findings. In the case of secondhand smoke exposure, this confirms previous research showing that those exposed to secondhand smoke have more severe ND [20, 21]. Taken together with the current findings, this suggests ND as a possible mechanism for why secondhand smoke exposure increases the risk of asthma. Future research is needed to disentangle the complex relationship between secondhand smoke exposure, ND, and asthma, including ND as a possible mediator.

Two potential explanations may underlie the current findings that smoking within 5 min of waking is associated with a heightened four-fold risk of asthma. Asthma involves inflammation and hypersensitivity of airways which could be a result of persistent irritation by the components of cigarette smoke [23]. Given that smokers can moderate their nicotine intake by altering their smoking style [24, 25], more dependent smokers may inhale more deeply and take more puffs per cigarette [26, 27]. If dependent smokers (as indicated by smoking within 5 min of waking) routinely extract more nicotine, and incidentally more smoke and tars from each cigarette, this may raise the risk of increased inflammatory mediators and bronchiolar damage. This could explain the significantly higher risk of lifetime asthma among such smokers. In this explanation, the effect of ND on asthma would be fully mediated through nuanced smoking style rather than the number of cigarettes consumed.

A second potential explanation could be that the relationship between ND and asthma may be the result of a common genetic predisposition. Genetic pathways have been identified that increase the risk of both ND and COPD [28, 29] as well as ND and lung cancer [30–32]. Though no such common genetic risk is currently known for ND and asthma, independent genetic risk factors have been identified for both ND [33–36] and asthma [37–39]. Future genetic studies examining potential *common* genetic links are warranted to test this potential explanation.

### Strengths and limitations

Strengths of this study include its novelty in establishing an association between smoking within 5 min of waking, a symptom of ND, and lifetime asthma. Additionally, the use of data from the large, nationally-representative NHANES survey increase the generalizability of our findings to current adult smokers in the US. The current study extends the existing literature in important ways by demonstrating a novel link between smoking within 5 min of waking, an indicator of ND, and asthma outcomes.

Limitations of the current study should be taken into account. First, due to the use of cross-sectional data, temporality and causation cannot be examined. This is especially important considering that asthma often develops in children who have not yet initiated smoking; however, the current study reduced this limitation by excluding participants whose asthma preceded smoking initiation. Second, the results are limited to the accuracy of self-reported variables. It is possible that some diagnoses of asthma are other types of pulmonary disorders such as chronic obstructive pulmonary disease, bronchitis, etc. However, the wording of the question (“have you ever been told by a doctor or health professional…”) reduces the likelihood of an incorrect diagnoses. Nevertheless, follow-up research is necessary to replicate these findings using clinical diagnosis rather than self-report. Third, the measurement of ND in NHANES is restricted to a single question (i.e. TTFC) to current smokers; this prevents an examination of ND among ex-smokers or passive smokers and excludes other aspects of the multi-dimensional construct of ND. Finally, the current study is intended as a preliminary examination of a novel association, and future research is needed to more rigorously test the relationship between ND and asthma outcomes.

## Conclusions

The current preliminary findings report a novel association between smoking within 5 min of waking, an indicator of ND, and lifetime occurrence of asthma. That is, among smokers with similar smoking histories, those who are more “addicted” as indicated by sooner smoking after waking have an approximately four-fold risk of having asthma at some time in their lives, independently of smoking behavior and other risk factors for asthma. This novel finding has important potential implications for treating smokers suffering from asthma. In particular, if the current preliminary findings are corroborated and extended in future research, healthcare professionals could screen smokers based on how soon they smoke after waking in order to more accurately assess their risk for asthma, among other health outcomes. Through more detailed risk assessment of smokers, closer monitoring and increased support for smoking cessation for those at risk for asthma, we could ultimately reduce the disease burden associated with asthma.

## Additional file

Additional file 1: **Table S1.** Unweighted regression results of smoking within 5 min of waking on asthma outcomes. (DOCX 16 kb)

## Abbreviations

COPD: Chronic Obstructive Pulmonary Disease
CPD: Cigarettes per Day
IDeA: Institutional Development Award
ND: Nicotine Dependence
NHANES: National Health and Nutrition Examination Survey
OR: Odds Ratio
SHS: Second Hand Smoke
TTFC: Time to First Cigarette
