# Supplementary material for: Earlier smoking after waking and the risk of asthma: a cross-sectional study using NHANES data
Source: BMC Pulm Med. 2018 Jun 18;18:102. doi: 10.1186/s12890-018-0672-y (PMC6006732; doi:10.1186/s12890-018-0672-y)
Supplement: Supplementary file 1 — Table S1. Unweighted regression results of smoking within 5 min of waking on asthma outcomes. (DOCX 16 kb) [file 12890_2018_672_MOESM1_ESM.docx]

**Additional file 1**

**Table S1: Unweighted regression results of smoking within 5 minutes of waking on asthma outcomes.**

| **Model** | **Covariate** | **Outcome** | | |
| --- | --- | --- | --- | --- |
|  |  | **Lifetime Asthma**  OR (95% CI)  *p-* value | **Past-Year Asthma**  OR (95% CI)  *p-*value | **Past-Year Asthma Attack**  OR (95% CI)  *p* value |
| Unadjusted | Smoking within 5 min. (vs. > 5 min.) | **1.62 (1.29-2.01)**  ***p* < .001** | **1.69 (1.32-2.19)**  ***p* < .001** | **1.63 (1.15-2.28)**  ***p* = .005** |
| Adjusted for smoking behavior^a^ | Smoking within 5 min. (vs. > 5 min.) | **1.45 (1.15-1.83)**  ***p* = .002** | **1.48 (1.13-1.93)**  ***p* = .004** | 1.34 (0.93-1.91)  *p* = .115 |
| Adjusted for smoking^a^ and other covariates^b^ | Smoking within 5 min. (vs. > 5 min.)  Smoking within 5 min x SHS  Smoking within 5 min x sex | **2.88 (1.07-7.23)**  ***p*=.028**  0.37 (0.14-1.04)  *p*=.050    **---------------------** | 2.42 (0.88-6.22)  *p*=.073  --------------------  0.96 (0.60 – 1.50)  *p* = .846 | 1.13 (0.70-1.83)  *p*=.610    --------------------  -------------------- |

Legend: Results are presented as odds ratio (95% confidence interval), *p*-value. Boldface indicates statistical significance (*p*<.05).

^a^ Smoking covariates: cigarettes per day and years of smoking duration.

^b^ Other covariates: depression, obesity, family history of asthma, secondhand smoke exposure (SHS), age, sex, race/ethnicity, and interactions of smoking within 5 minutes with secondhand smoke exposure and sex. Interaction terms are only included in the model if *p*<.10.
